# *Dientamoeba fragilis* cases identified by molecular detection, Utah, United States, 2014–2024

**DOI:** 10.1017/S0950268825000159

**Published:** 2025-02-07

**Authors:** Anna Jones, Marc Roger Couturier, Andrew T. Pavia, Daniel T. Leung

**Affiliations:** 1 Department of Pediatrics, University of Utah School of Medicine, Salt Lake City, UT, USA; 2 Department of Pathology, University of Utah, Salt Lake City, UT, USA; 3 Institute for Clinical and Experimental Pathology, ARUP Laboratories, Salt Lake City, UT, USA; 4 Division of Pediatric Infectious Disease, Department of Pediatrics, University of Utah School of Medicine, Salt Lake City, UT, USA; 5 Division of Infectious Disease, Department of Internal Medicine, University of Utah School of Medicine, Salt Lake City, UT, USA

**Keywords:** diarrhea, case series, protozoan, United States, PCR

## Abstract

*Dientamoeba fragilis* (*D. fragilis*) is an intestinal protozoan parasite with uncertain pathogenic potential. In the United States, data on *D. fragilis* in the era of molecular detection are limited. The aim of this retrospective chart review was to evaluate the epidemiology and clinical characteristics of *D. fragilis* cases identified using polymerase chain reaction assays between 2016 and 2024 at our academic medical centre located in Utah. We identified 28 unique cases with varying gastrointestinal symptomatology including diarrhoea, abdominal pain, nausea, vomiting, and bloating. Approximately half (52%) of patients with follow-up data demonstrated improvement in symptoms following initial treatment for *D. fragilis.* The overall prevalence of *D. fragilis* was low among those tested (0.6% positivity). Additional research, including case-control studies, is needed to better describe the etiologic role of *D. fragilis.*

*Dientamoeba fragilis* (*D. fragilis*) is an intestinal protozoan with unclear pathogenic potential [1–3]. *D. fragilis* is commonly reported in association with gastrointestinal (GI) symptoms but has also been commonly detected in asymptomatic persons [2,4,5]. *D. fragilis* is frequently detected with other organisms, complicating efforts to understand its pathogenicity [5,6]. The life cycle and transmission of *D. fragilis* are not completely understood, and multiple hypotheses exist to explain the protozoan’s presence in human GI tracts given the fragile nature of the trophozoite stage [7,8]. It has appropriately been called ‘a neglected protozoan’ [2,4]. The reported prevalence of *D. fragilis* varies depending on geographic location, study population, and diagnostic methods [2–4]. Additionally, the clinical presentation ranges from asymptomatic carriage to diarrhoea, abdominal pain, and peripheral eosinophilia [4–6]. With the increasing availability of molecular diagnostic methods, the identification of *D. fragilis* has been facilitated by use of both single- and multiplex polymerase chain reaction (PCR) assays, which have a significantly higher sensitivity than microscopy [3]. The majority of recent clinical and epidemiologic studies characterizing *D. fragilis* have been conducted in Europe [3,4], with the most recent study in the United States (US) being a microscopy-based study published over a decade ago [9]. At the time of this writing, only one FDA-cleared PCR assay is available from Genetic Signatures, and this product has been used in Australia and Europe with excellent performance [10]. Our primary objective was to describe the epidemiologic and clinical characteristics of PCR-diagnosed *D. fragilis* patients by performing a retrospective chart review at our academic medical centre located in the US.

The University of Utah has used the GI Parasite Panel by PCR developed by ARUP Laboratories since October 2014. The panel includes *Cryptosporidium hominis* and *parvum, Cyclospora* spp.*, Giardia, Entamoeba histolytica*, and *D. fragilis* targets. The *D. fragilis* target is a conserved sequence within the 18S rRNA gene. The analytical sensitivity is approximately 16,000 copies/ml of stool (equal to approximately 200 copies per reaction). Analytical specificity was established for each of the protozoal targets against each other and 42 additional viral, bacterial, and parasitic organisms (including *Entamoeba* spp. and *Strongyloides*). In silico analysis revealed no predicted cross-reactivity with other organisms, including all formally sequenced protozoa. All specimens were frozen immediately after collection and thawed only at the time of testing. This frozen stability was shown in validation to preserve sensitivity consistent with testing fresh stool. ARUP Laboratories recommend use of the panel for individuals with chronic diarrhoea and a travel history or other relevant exposure history or those with a complicated clinic course; the decision to order the test is ultimately left to the clinician [11].

Since the GI Parasite Panel by PCR became available, 4,804 tests have been performed on patients within the University of Utah Health system. The total positivity for any target is 181 (3.8%). For our report, a case of *D. fragilis* was defined by a positive PCR test; a patient with multiple positive PCR results was described as one case if there was no intervening negative result. We reviewed the charts of the *D. fragilis* cases to abstract relevant demographic and clinical data. Study data were collected and managed using REDCap electronic data capture tools hosted at the University of Utah [12,13]. This study was deemed exempt from full review by the University of Utah IRB (IRB_00101686).

Thirty-one samples were positive for *D. fragilis* (0.6% positivity). Of those 31, we identified 28 unique cases of *D. fragilis*, detected between April 2016 and April 2024. At least one case was identified each year, except for 2021. Apart from two cases, all patients were diagnosed in the outpatient setting, with most patients evaluated and treated in primary care clinics (Table 1). Several patients were diagnosed by gastroenterology and infectious disease specialists. The two hospitalized patients had underlying conditions, and their level of acuity was likely unrelated to the *D. fragilis* infection. One hospitalized individual was a bone marrow transplant recipient with concern for graft-versus-host disease as a possible aetiology of their presentation and the second was a patient with septic shock in the setting of a newly diagnosed HIV infection and multiple co-infections.

**Table 1.** Demographic characteristics of cases

	n (%)
Total cases	28
Female sex	17 (61)
Male sex	11 (39)
Median age	33
<5 years	0 (0)
5–17 years	5 (18)
18–49 years	17 (61)
50+ years	6 (21)
Encounter type	
Clinic	23 (82)
Hospital	2 (7)
Other	3 (11)
Insurance type	
Private	20 (71)
Other	8 (29)
Provider specialty	
Primary care	15(54)
Infectious disease	5 (18)
Gastroenterology	4 (14)
Other	4 (14)
History of international travel	
Yes^a^	11 (39)
No	6 (21)
Unknown	11 (39)
Immunocompromised state	
Yes	3 (11)
No	25 (89)

a Destinations visited: Columbia, Japan, Madagascar, Malawi, Mexico (4), Pacific Islands, Peru (3), Philippines, Puerto Rico, Singapore, Spain, and Vietnam.

At the time of data abstraction, 25 patients had addresses in urban Utah counties and 3 were from urban counties in nearby states. The median age was 33; 17 (61%) patients were between the ages of 18 and 49 years (Table 1). Seventeen (61%) were female. Eleven (39%) individuals reported a history of recent international travel. An additional two individuals (7%) had a history of freshwater exposure in the US. Most individuals presented with persistent GI symptoms, and several with greater than 1 year of symptoms (Table 2), and most had multiple GI complaints (79%). Approximately 82% of patients reported diarrhoea. Abdominal pain (61%), nausea (46%), bloating (39%), and constipation (25%) were also common.

**Table 2.** Reported symptoms

	n (%)
Median length of symptoms in days (min, max)^a^	45 (3, 700)
Reported diarrhoea	23 (82)
3 or more loose stools per day	9 (32)
Blood in stools	3 (11)
Abdominal pain	17 (61)
Nausea	13 (46)
Vomiting	6 (21)
Bloating	11 (39)
Constipation	7 (25)
Subjective fever	5 (18)
Objective fever	0 (0)
Weight loss	4 (14)
Anorexia	4 (14)
Fatigue	4 (14)
Anal pruritus	4 (14)
Multiple gastrointestinal complaints	22 (79)

a Missing in 4 cases.

Enteric co-detections were not commonly identified. Twenty-five (89%) cases had infectious diarrhoea testing in addition to the GI Parasite Panel PCR (Table 3). One patient was also positive for astrovirus (identified by comprehensive GI pathogen PCR panel), and another individual was positive for *Blastocystis* (identified by stool ova and parasite testing). A third patient was newly diagnosed with HIV and was also positive for *Shigella* and EPEC (also identified by GI pathogen PCR panel). In the ten patients with ova and parasite (O&P) examination results, none were positive for *D. fragilis.* In the ten patients with CBC results, one (10%) demonstrated eosinophilia; this was the aforementioned patient with recently diagnosed HIV and *Shigella* and EPEC co-detections. An additional patient was evaluated due to history of persistent eosinophilia and ultimately was diagnosed with systemic mastocytosis, a likely contributor to the eosinophilia.

**Table 3.** Additional infectious diarrhoea testing. Additional testing was performed on 25 (89%) cases

	Testing ordered n (% of cases)	Test positivity n (% of tests ordered)
Comprehensive GI pathogen PCR panel	2 (7)	2 (100)
Stool viral PCR panel	5 (18)	0 (0)
Stool bacterial PCR panel	4 (14)	0 (0)
Stool culture	12 (43)	0 (0)
Stool ova and parasite	10 (36)	1 (10)
*C. difficile* toxin by EIA	14 (50)	0 (0)
*Campylobacter* antigen	8 (29)	0 (0)
*Helicobacter pylori* antigen	5 (18)	0 (0)
*Strongyloides* antibody	4 (14)	0 (0)
*Schistosoma* antibody	1 (4)	0 (0)
*Giardia* antigen	1 (4)	0 (0)
Pinworm	1 (4)	0 (0)

All individuals were treated for *D. fragilis.* The majority were prescribed metronidazole (89%) as initial treatment. One individual was prescribed paromomycin, another individual was prescribed tinidazole due to a history of multiple rounds of metronidazole for *Blastocystis* treatment, and a third was treated for concomitant chlamydia infection with doxycycline. In the 25 cases with follow-up data available, symptoms improved in 13 (52%) after one round of treatment. Seven (26%) patients were retested due to persistent symptoms following treatment; only two remained positive for *D. fragilis* tests upon retesting (Supplemental Table 1). Four (15%) received additional rounds of treatment with either metronidazole or doxycycline; none of those who received additional rounds of treatment experienced a resolution of symptoms.

In this single-centre retrospective study of PCR-positive *D. fragilis* cases over a 10-year period of PCR testing availability, we found an overall test positivity rate of 0.6%. Prior prevalence estimates vary considerably based on geographic region, population studied, and diagnostic method employed [2–4]. Our positivity rate was higher than a 2010 study of intestinal infections in the Rocky Mountain region, which found a 0.04% prevalence of *D. fragilis* identified using microscopy [14] and notably lower than the reported prevalence of *D. fragilis* identified using PCR in symptomatic individuals in European countries and Australia [2,5,15]. Due to the limited availability of *D. fragilis* PCR in the US, the clinical presentation and treatment outcomes of patients with *D. fragilis* in the US are not well known.

Testing was requested only on symptomatic individuals; without a control group, we cannot clearly attribute *D. fragilis* as the cause of the symptoms. Additional viral or bacterial testing was documented on most (89%) patients. Most patients (88%) with additional testing had *D. fragilis* identified as a single organism. However, three had a co-detection documented and we identified alternative diagnoses through chart review in two (irritable bowel syndrome and systemic mastocytosis). The scarcity of co-detections and alternative diagnoses is a strength of our case series as these have limited the ability to understand the pathogenicity of *D. fragilis* [6,16].

The range of GI symptoms of the patients in our study was similar compared to other studies [2,16,17]. Interestingly, only 10% had eosinophilia, which differs from prior reports [2,5,16,18], though only approximately one-third of patients had CBC results for evaluation. Additionally, among the one-third of cases which also had an O&P examination performed, none were positive for *D. fragilis.* This is not unexpected given the high sensitivity of PCR and challenging nature of direct microscopy [19].

This study may have limited generalizability due to the single centre of data collection. Additionally, all patients in our review were tested due to the presence of GI symptoms, limiting our ability to draw conclusions about the etiologic role of *D. fragilis.* It is possible that other underlying causes, such as IBS, may contribute to symptomatology seen in patients in whom *Dientamoeba* is detected. The lack of follow-up data in this retrospective study limits our assessment of treatment efficacy.

We found that among patients from the Intermountain West who were tested using a multiparasite PCR assay, the prevalence of *D. fragilis* was low. Case-control studies in the US could help determine the prevalence among asymptomatic persons and better describe the etiologic role of *D. fragilis.* The reasons for the low prevalence in this sample of US patients compared to the prevalence in Europe require further study.

## Supporting information

Jones et al. supplementary material Jones et al. supplementary material

## Data Availability

Deidentified data that support the findings of this study are available from the corresponding author upon reasonable request.

## Supplementary material

For supplementary material accompanying this paper visit http://doi.org/10.1017/S0950268825000159. click here to view supplementary material
