# Supplementary material for: Dientamoeba fragilis cases identified by molecular detection, Utah, United States, 2014–2024
Source: Epidemiol Infect. 2025 Feb 7;153:e36. doi: 10.1017/S0950268825000159 (PMC11869066; doi:10.1017/S0950268825000159)
Supplement: Jones et al. supplementary material [file S0950268825000159sup001.docx]

**Supplemental Table 1.** Outcomes of cases that were re-tested.

| Case | Outcome |
| --- | --- |
| Female, age category 18-49 years, with concern for pinworms and anal pruritus | Treated with metronidazole. Re-testing due to persistent symptoms was negative. No additional *D. fragilis* treatment. |
| Female, age category 18-49 years, with diarrhea and nausea | Treated with metronidazole and then doxycycline due to concern for treatment failure. Retesting after doxycycline was negative. No additional *D. fragilis* treatment. |
| Female, age category 5-17 years with diarrhea, abdominal pain, nausea, and bloating | Treated with metronidazole. Re-testing due persistent symptoms was negative. No additional *D. fragilis* treatment. Ultimately diagnosed with IBS. |
| Female, age category >50 years, with diarrhea, constipation, bloody stools, and bloating | Treated with metronidazole. Re-testing due persistent symptoms was positive. Treated with second round of metronidazole. Additional testing was negative. Patient still with symptoms. |
| Male, age category 18-49 years, with diarrhea, constipation, bloating, weight loss, and fatigue | Treated with metronidazole. Re-testing due persistent symptoms was negative. No additional *D. fragilis* treatment. |
| Male, age category 18-49 years, with diarrhea, constipation, nausea, vomiting, and bloating | Treated with metronidazole. Re-testing due persistent symptoms was positive. Treated with doxycycline. Re-tested after doxycycline was negative. No additional *D. fragilis* treatment. Patient still with persistent symptoms. |
| Female, age category 18-49 years, with diarrhea, constipation, abdominal pain, and bloating | Treated with metronidazole. Re-testing due persistent symptoms was negative. No additional *D. fragilis* treatment. Diagnosed with small intestinal bacterial overgrowth. |
